# Generation and Breeding of *EGFP*-Transgenic Marmoset Monkeys: Cell Chimerism and Implications for Disease Modeling

**DOI:** 10.3390/cells10030505

**Published:** 2021-02-27

**Authors:** Charis Drummer, Edgar-John Vogt, Michael Heistermann, Berit Roshani, Tamara Becker, Kerstin Mätz-Rensing, Wilfried A. Kues, Sebastian Kügler, Rüdiger Behr

**Affiliations:** 1 Platform Degenerative Diseases, German Primate Center–Leibniz Institute for Primate Research, Kellnerweg 4, 37077 Göttingen, Germany; ejvogt74@gmail.com; 2 DZHK (German Center for Cardiovascular Research), Partner Site Göttingen, 37099 Göttingen, Germany; 3 Endocrinology Laboratory, German Primate Center–Leibniz Institute for Primate Research, Kellnerweg 4, 37077 Göttingen, Germany; MHeistermann@dpz.eu; 4 Unit of Infection Models, German Primate Center–Leibniz Institute for Primate Research, Kellnerweg 4, 37077 Göttingen, Germany; broshani@dpz.eu; 5 Primate Husbandry, German Primate Center–Leibniz Institute for Primate Research, Kellnerweg 4, 37077 Göttingen, Germany; tbecker@dpz.eu; 6 Pathology Unit, German Primate Center–Leibniz-Institute for Primate Research, Kellnerweg 4, 37077 Göttingen, Germany; kmaetz@dpz.eu; 7 Friedrich-Loeffler-Institut, Institut für Nutztiergenetik, Mariensee, 31535 Neustadt, Germany; wilfried.kues@fli.de; 8 Center for Nanoscale Microscopy and Physiology of the Brain (CNMPB) at Department of Neurology, University of Göttingen, Waldweg 33, 37073 Göttingen, Germany; sebastian.kuegler@med.uni-goettingen.de

**Keywords:** marmoset monkey, non-human primate, embryo, genetic modification, germline transmission, germ cell, transgenesis, chimerism, hematopoietic stem cell

## Abstract

Genetic modification of non-human primates (NHP) paves the way for realistic disease models. The common marmoset is a NHP species increasingly used in biomedical research. Despite the invention of RNA-guided nucleases, one strategy for protein overexpression in NHP is still lentiviral transduction. We generated three male and one female enhanced green fluorescent protein (EGFP)-transgenic founder marmosets via lentiviral transduction of natural preimplantation embryos. All founders accomplished germline transmission of the transgene by natural mating, yielding 20 transgenic offspring together (in total, 45 pups; 44% transgenic). This demonstrates that the transgenic gametes are capable of natural fertilization even when in competition with wildtype gametes. Importantly, 90% of the transgenic offspring showed transgene silencing, which is in sharp contrast to rodents, where the identical transgene facilitated robust EGFP expression. Furthermore, we consistently discovered somatic, but so far, no germ cell chimerism in mixed wildtype/transgenic litters. Somatic cell chimerism resulted in false-positive genotyping of the respective wildtype littermates. For the discrimination of transgenic from transgene-chimeric animals by polymerase chain reaction on skin samples, a chimeric cell depletion protocol was established. In summary, it is possible to establish a cohort of genetically modified marmosets by natural mating, but specific requirements including careful promoter selection are essential.

## 1. Introduction

Non-human primates (NHP) reflect human anatomy, physiology, and genetic constitution much better than other animal species [1,2]. This is of relevance in many research areas including neurobiology [2,3,4,5,6,7,8], reproductive biology [9,10,11], and genetics [11,12,13,14]. Only by adding NHP to the repertoire of experimental animal models, specific characteristics of NHP and human organismic physiology and pathology can be studied adequately [15,16]. Thus, NHP are indispensable for preclinical translational studies involving the development and testing of novel treatment options using, e.g., stem cells [17,18,19,20,21] and biologics [1,22], in the context of the whole organism.

Major challenges of translational biomedical research are effective treatments of currently untreatable diseases including neurodegenerative, cardiovascular, and metabolic disorders. Many of them are caused or promoted by the mutation of specific genes [23,24,25,26,27]. However, although millions of people are affected by these diseases, access to diseased human tissue, particular from relevant *pre mortem* stages, is extremely limited. Moreover, experimental therapeutic studies in humans, who are not terminally ill, are unethical. These facts severely hamper progress in the battle against many diseases. Since the spontaneous occurrence of many diseases in NHP has not been observed so far, genetic modification of NHP is a promising approach to generate realistic and meaningful animal models to better understand and eventually treat human diseases and to illuminate primate biology. Lentiviral transgenesis, which has been shown to be highly efficient and robust in mice and rats [28], is a feasible strategy to also generate genetically modified (GM) NHP. Lentiviral enhanced green fluorescent protein (EGFP) transgenesis of NHP has been achieved in proof of concept studies using marmosets (*Callithrix jacchus*) [29] and macaques (*Macaca mulatta* and *Macaca fascicularis*) [30,31,32]. Also, marmoset [33,34] and macaque [35,36,37] disease models were generated by lentiviral transgenesis. Recently, marmoset transgenesis also provided novel insights into primate brain evolution [38]. However, to establish a cohort of naturally conceived NHP with identical genetic modifications to conduct controlled studies in the future, germline transmission of a disease- or phenotype-inducing transgene to offspring is essential. Only relatively few NHP studies, however, reported germline transmission of a transgene and its propagation to offspring so far, and the number of living transgenic offspring was rather low. Although Liu et al. [36] and Moran et al. [39] used cynomolgus and rhesus macaques, respectively, Sasaki et al. [29], Tomioka et al. [33,34] and Park et al. [40] used the marmoset monkey. Most studies employed assisted reproductive technologies (ART) [29,33,34,36,39,40] including intracytoplasmic sperm injection (ICSI) [33,34,36] and in vitro fertilization (IVF) [29,40] to achieve germline transmission. Only Tomioka and colleagues reported transmission of a transgene through the germline from the mother to one pup by natural mating in marmosets [33].

Macaques and the marmoset monkey differ in relevant biological characteristics [41]. Although rhesus monkeys are phylogenetically closer to humans than marmoset monkeys [42], the latter species has invaluable practical advantages for long-term trans-generational studies. These include a shorter generation time (1.5–2 years versus 4–6 years in macaques), a greater fecundity of marmosets (marmosets: two litters with usually 2–3 pups twice a year; macaques: singleton birth once per year), a lack of reproductive seasonality, and a lack of zoonoses, which can severely complicate work with macaques [43]. Therefore, marmoset studies are significantly more time and cost-efficient than studies with macaques.

In the present study we generated four fertile *EGFP*-transgenic founder marmosets. We report efficient transmission of the transgene to offspring through the male and the female germline by natural mating. Importantly, in sharp contrast to rodents harboring the identical transgene [28], the transgene was silenced during germline transmission in 18 out of 20 (90%) transgenic F1 marmosets in the present study. Furthermore, analysis of the progeny revealed consistent somatic cell micro-chimerism between littermates. Our data highlight a high frequency of transgene silencing during germline transmission in marmoset monkeys as well as the risk of false-positive genotyping results in litters consisting of transgenic and non-transgenic (chimeric) animals. Regarding the latter, we provide a protocol to prevent the false-positive genotyping from skin biopsies.

## 2. Materials and Methods

### 2.1. Animals and Animal Housing

All experiments were carried out in accordance with relevant guidelines. Marmoset monkeys (*Callithrix jacchus*) for this study were obtained from the self-sustaining breeding colony of the German Primate Center (Deutsches Primatenzentrum; DPZ). This study, including ethics, was approved by the competent authority, the regional government office, under license numbers AZ 42502-04-10/0063 and AZ 42502-04-14/1652. Housing of GM marmoset monkeys was approved according to the German *Gentechnikgesetz* by the *Staatliches Gewerbeaufsichtsamt Göttingen* (AZ 40611/0505/505 GOE023278161). The legal guidelines for the use of animals and the institutional guidelines of the DPZ for the care and use of marmoset monkeys were followed. Where applicable, the ARRIVE (Animal Research: Reporting of In Vivo Experiments) guidelines were followed. Health and well-being of the animals were controlled daily by experienced animal care attendants and regularly, at least twice per week, by veterinarians. All interventions were performed by experienced veterinarians or by trained assistance personnel under the supervision of veterinarians. Marmoset monkeys are social tree-living New World monkeys originating from the tropical northeast of Brazil. Accordingly, the animals were pair-housed in a temperature- (25 ± 1 °C) and humidity-controlled (65 ± 5%) facility. These parameters were controlled daily. Room air was changed several times per hour and filtered adequately. Illumination was provided by daylight and additional artificial lighting on a 12.00:12.00 h light:dark cycle. Each cage consisting of stainless steel had a vertical orientation [165 cm (height) × 65 cm (width) × 80 cm (depth)] and was furnished with wooden branches and shelves for environmental enrichment and contained a wooden sleeping box mimicking the monkeys’ natural habitats. The housing room and the cages were cleaned with water at weekly intervals. The animals were fed ad libitum with a pelleted marmoset diet (ssniff Spezialdiäten, Soest, Germany). In addition, 20 g mash per animal was served in the morning and 30 g fruit or vegetables mixed with noodles or rice were supplied in the afternoon. Furthermore, once per week mealworms or locusts were served to provide adequate nutrition. Drinking water was always available. All materials were changed regularly, cleaned, and sterilized.

### 2.2. Embryo Collection

Female common marmosets (*n* = 20) in the age range of 2.3 to 11.75 years were used as embryo donors and 13 females in the age range of 4.3 to 13.2 years as embryo recipients (surrogate mothers). All embryo donors were kept pairwise with fertile males, and their reproductive cycles were monitored by blood sampling to determine progesterone concentrations [44]. Marmosets in the luteal phase received synthetic prostaglandin F2α (0.2 mL of a mixture of 0.1 mL Estrumate^®^ and 3.2 mL Ringer lactate solution) in order to reinitiate the reproductive cycle starting with the follicular phase [45]. This allowed timing of ovulation. Individualized blood sampling schemes were applied to each monkey to reduce the number of samplings without losing precision in the determination of ovulation. Collection of natural embryos was performed by flushing the uterus on day four to seven after ovulation to obtain early preimplantation stages consisting of as few cells as possible, i.e., morula stages of ~ 8–32 cells. Earlier stages of natural embryos cannot be obtained since these stages are still inaccessibly present in the oviduct. The uterus was flushed either by invasive, minimal-invasive, or non-invasive methods. Invasive and minimal-invasive embryo collection was performed as described previously [46]. The non-invasive trans-vaginal embryo collection approach was similar to the one described previously [47].

### 2.3. Lentiviral Vector Production

The lentiviral FUGW vector was produced in 293-FT cells by transient transfection with pCMV-dR8.2 (Addgene plasmid 8455), pVSV.G (Addgene plasmid 14888) and pFUGW (Addgene plasmid 14883), which was a gift from David Baltimore [28]. Cells were grown in 4-level cell factories (Thermo Scientific, Langenselbold, Germany, # 140004), transfected overnight, grown in 800 mL DMEM with 10% FCS for 24 h, then the medium was changed to 200 mL serum-free OptiPRO medium (Life Technologies) for another 24 h. The cell culture supernatant was centrifuged for 10 min at 800× *g*, filtered through a 0.22 µM filter (Millipore Express Plus) and centrifuged two times through a 20% sucrose cushion (80,000× *g*, 2 h 4 °C). The final pellet was resuspended in 50–75 µL phosphate buffered saline (PBS), cleared from any remaining particular matter by centrifugation at 800× *g* and stored at −80 °C until use. The viral vector expressed EGFP under control of the ubiquitous human ubiquitin C (UBC) promoter [48]. The functional titer was determined by transduction of terminally differentiated primary cortical neurons in serial dilutions and was routinely about 1.5 × 10^10^ transducing units per mL.

### 2.4. Microinjection of a Lentiviral Vector Encoding for EGFP

Embryos for lentiviral injections were transferred into a 20 µL drop M2 medium (M7167, Sigma-Aldrich, St. Louis, MO, USA) supplemented with 0.25 M sucrose. The medium was covered with embryo culture-tested oil (Irvine Scientific, Santa Ana, CA, USA). The hyperosmolar sucrose solution induced shrinkage of the cells of the embryo resulting in the formation of an expanded perivitelline space, which was sufficient for the injection volume of the lentiviral suspension. The virus suspension with a titer of 5 × 10^8^ transducing units/mL was injected through the *zona pellucida* with a pressure of 150 to 195 hPa. Immediately after injection, the embryos were transferred to surrogate mothers. Control embryos were kept in culture for up to 48 h to check for the timing of EGFP induction.

### 2.5. Embryo Transfer

Breeding-experienced proven mothers, who were kept together with sterilized males, were used as embryo recipients. The embryo recipient’s reproduction cycle was monitored by serum progesterone measurements, and embryo donor’s and recipient’s reproductive cycles were synchronized by prostaglandin F2α injection. Before embryo retransfer, the recipient received an i.m. injection of short-term anesthetics (Diazepam 0.05 mL/animal and Alfaxan 0.1 mL/100 g bodyweight). The catheter was inserted transvaginally under ultrasound guidance into the uterus. One to four embryos were transferred in a maximum volume of 1.5 µL medium. Then the embryos were expelled under ultrasonography control (GE logiq e). Awakening of the animal was supported by a red-light warming lamp positioned above the single cage in an undisturbed environment. Embryo recipients were supplemented orally with the synthetic gestagene Altrenogest (Regumate^®^, Intervet, Unterschleißheim, Germany) daily for ten days. Pregnancies were diagnosed biochemically by the determination of progesterone in blood samples (constantly elevated above 10 ng/mL) from day 28 after embryo retransfer onwards and by ultrasound inspection of the implantation sites. For control purposes of the effect of the virus injection and integration, also uninjected embryos were retransferred to recipients after their retrieval. These embryos were treated in the same way as the injected embryos except that the injection procedure was omitted.

### 2.6. Skin Biopsy, Blood, and Sperm Sampling for Expression Analysis

Skin biopsies were performed under short-term anesthesia with Diazepam (0.05 mL i.m./animal) and Alfaxan (0.1 mL i.m./100 g bodyweight). A piece of skin (approximately 4 × 8 mm) was taken from the lateral abdominal wall and the incision closed by an intra-cutaneous suture of the superficial skin. Blood (~0.2 mL) for *EGFP* analysis was taken from the *Vena femoralis*. Sperm was collected from awake animals by penile vibrostimulation using a vibrator (Ferticare^®^, Multicept, Rungsted, Denmark) modified with a silicon rubber holder for collecting tubes. The vibrating tube was placed onto the penis and a stimulation protocol with defined amplitudes and intensity was run [49].

### 2.7. Cell Culture of Primary Fibroblasts

Skin fibroblasts were isolated and cultured as previously described [50]. Images were taken using a Zeiss Cells Observer equipped with the Zeiss Axiovision 4.8.2.0 software version (Jena, Germany).

### 2.8. PCR Analysis of Genomic DNA

Two samples of each animal, skin and blood cells, were used as separate DNA sources for genotyping. Where possible, DNA from fibroblasts that were cultured (see above) for several days was also isolated as a third sample. Genomic DNA was isolated using the DNeasy kit (#69504, Qiagen^®^, Hilden, Germany). PCR was performed according to standard procedures running 32 cycles. Primers used for the amplification of *β-Actin* were forward 5′-GACGACATGG AGAAGATCTG G-3′ and reverse 5′-GGAAAGAAGGCTGGAAGAGT G-3′. The *EGFP* fragment was amplified using forward primer 5′-CTACCTGAGCACCCAGTCCG-3′ and reverse primer 5′-AAAGGAGCAACATAGTTAAGAATAC-3′ or alternatively with forward primer 5′-CGCTATGTGGATACGCTGCTT-3′ and reverse primer 5′-CAG CCAAGGAAAGGACGATGA-3′. The gels were imaged using an Intas Gel ix Imager equipped with the Intas GDS Software (INTAS Science Imaging Instruments, Göttingen, Germany).

### 2.9. Western Blot

For western blotting, the cytoplasmic and nuclear protein fractions of fibroblasts were prepared using the Qproteome nuclear protein kit (#37582, Qiagen^®^, Hilden, Germany). The beta-Actin antibody was from Santa Cruz Biotechnology, Dallas, Texas, USA (#SC 1616-R) and the EGFP antibody was from Abcam (Cambridge, UK; # ab290). β-Actin and EGFP were detected using 1:1000 and 1:3000 dilution, respectively. The secondary HRP-conjugated anti-rabbit antibody (R&D Systems, Wiesbaden, Germany, #HAF008) was used at a 1:1000 dilution. Signals were visualized using the chemoluminescence ECL Western Blotting Analysis System (GE Healthcare, Chicago, IL, USA, RPN2108) and the Intas ChemoCam Western Blot Imaging System equipped with the Chemo Star Professional Software (INTAS Science Imaging Instruments, Göttingen, Germany).

### 2.10. Flow-Cytometric Analysis

Flow-cytometric analyses (including negative and positive controls) were performed several times with fibroblasts, whole blood, and sperm resulting in highly comparable results. 2.5–5 × 10^5^ fibroblasts were fixed in 4% formaldehyde in PBS for 7 min before measurement. 50 μL of whole blood were incubated with 1 mL red blood cell (RBC) lysis/fixation solution (BioLegend, San Diego, CA, USA) for 15 min to lyse residual RBCs and to fix cells. Whole ejaculates were suspended in 100 µL PBS. To exclude dead cells, the suspended ejaculates were stained for 15 min using the live/dead fixable violet dead cell stain kit (Invitrogen, Karlsbad, CA, USA) according to manufacturer’s instructions. Following a washing step with PBS supplemented with 0.5% BSA, cells were analyzed for EGFP using a custom-made LSRII cytometer (BD Biosciences, Heidelberg, Germany) equipped with three lasers. Analysis of EGFP expression was done using FlowJo 9.9.4 (Treestar, Ashland, OR, USA). The live/dead staining in combination with an appropriate gating strategy were used to exclude dead cells, doublets, round cells, and debris from the analyses of spermatozoa. Accordingly, the forward scatter (FSC) and side scatter (SSC) voltages were set to measure only mature spermatozoa. The analyses of spermatozoa shown in Figures 5C (founder) and 6G (F1 animals) were performed in one run and the spermatozoa analysis from wildtype animal #16 is shown as a control in both figures for the purpose of clarity.

### 2.11. Immunohistochemistry

Immunohistochemistry was generally performed as described previously [51] with an additional tissue section digestion by Proteinase K (50 µg/mL; Sigma-Aldrich P 6556) for 10 min. Then the sections were stained using an immunohistochemical staining kit (DakoCytomation Carpinteria, CA, USA, LSAB+ system-HRP, K0679) and either the anti-EGFP-antibody from Abcam (ab290) in a 1:800 dilution or from cell signaling (255S) in a 1:500 dilution.

### 2.12. Genomic Southern Blot Analysis

Genomic DNA was isolated by lysing 50 mg of tissue with lysis buffer (100 mM TRIS-HCL pH 8.5; 0.2% SDS; 5 mM EDTA; 200 mM NaCl) and Proteinase K (10 mg/mL) and incubated overnight at 55–60 °C in a shaker. After centrifugation (15 min at 21,000× *g*) 560 µL of saturated NaCl (5.5 M) was added to 400 µL of the supernatant followed by another centrifugation step (15 min with 14,000 RPM) for protein precipitation. 700 µL of 100% ethanol was added to the supernatant and mixed thoroughly for DNA precipitation. The pellet was washed several times in 70% ethanol, dried at room temperature and resuspended in 50 µL H_2_O at 37 °C overnight. DNA was diluted if the concentration was above 2 µg/µL. After digestion with NcoI, the DNA was then electrophoresed, blotted, and hybridized to a DIG-labeled probe against EGFP. After washing, the probe was visualized using a chemiluminescence kit (Roche Diagnostics, Mannheim, Germany) and a Vilber Lourmat Fusion-SL3500 documentation system. Flanking DNA fragments were expected to be >3 kb.

### 2.13. Statistics

The difference in the birth rates between the embryo groups (injected vs. natural embryos) was tested by an analysis of a 2 × 2 contingency table with the Fisher’s exact test. Differences in average body weight were tested using the Mann-Whitney-U-Test.

## 3. Results

### 3.1. Generation of EGFP-Positive Founder Animals

Natural marmoset embryos retrieved from the uterus have a better developmental potential than in vitro fertilized embryos [29]. Therefore, we used natural preimplantation embryos instead of IVF embryos, and injected them with lentiviral vector encoding EGFP under the control of the human ubiquitin C promoter, which was previously shown to mediate stable transgene expression in founders and F1 progeny of mice [28,48] and rats [28].

**Figure 1:**
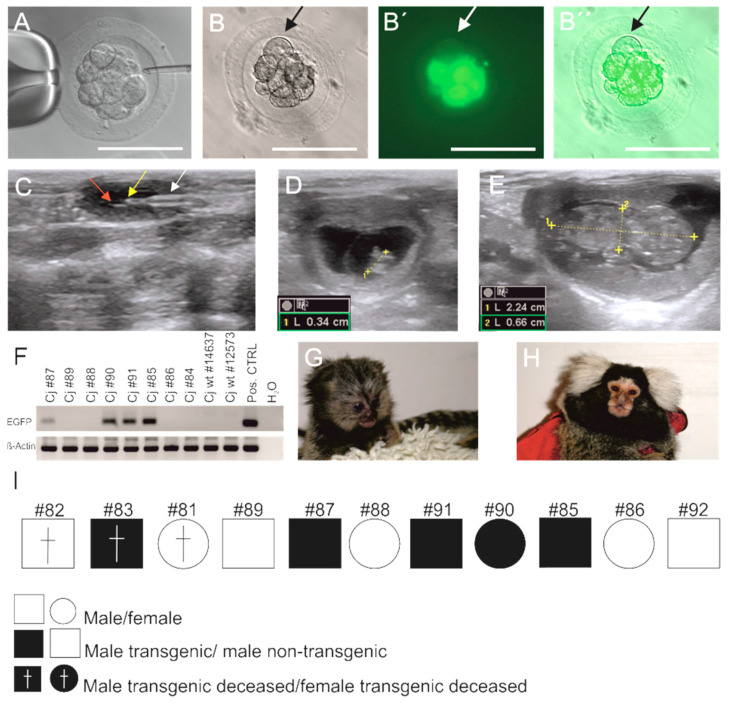
Generation of EGFP-transgenic marmoset monkeys. (**A**) Injection of the virus suspension into the perivitelline space of an 8–16 cell embryo. Scale bar: 100 µm. (**B**) Bright-field image of an EGFP virus-injected embryo. (**B′**) Fluorescent image of the embryo shown in (**B**)), 4 h after virus injection. The arrows highlight a blastomere lacking green fluorescence. (**B″**) Merged images of (**B**) and (**B′**). (**C**) Ultrasonographic image of an embryo transfer (ET) into a surrogate mother. The transfer catheter (white arrow) is placed in the lumen of the uterus (red arrow; yellow arrow: embryo). (**D**) Ultrasonographic image of an embryo 49 days after ET and (**E**) of an embryo 85 days after ET. (**F**) PCR genotyping of monkeys obtained after virus injection and ET. Animals #87, #90, #91, and #85 were EGFP-positive. (**G**) A transgenic postnatal marmoset (#90). (**H**) A transgenic adult marmoset (#90). (**I**) Overview of the postnatal animals obtained from injected embryos.

Initially, we injected 5 embryos to demonstrate effective transduction of the individual blastomeres in marmoset monkey embryos (Figure 1A). EGFP fluorescence was detectable in vitro already four hours after injection (Figure 1B,B′,B″). Although most cells of the embryo showed strong EGFP signals, some cells were negative (Figure 1B,B′,B″; arrows).

Then we used 113 embryos for virus injection (Table 1) of which 110 were transferred to surrogate mothers during 46 embryo transfers (ET) (Figure 1C). Fourteen initiated pregnancies confirmed by progesterone measurements and ultrasound (Figure 1D,E) eventually resulted in the birth of 11 neonates (Table 1) with normal progression of intrauterine development and gestation periods as monitored by ultrasound [51,52]. The birth rate/ET was 23.9% (Table 1).

**Figure 2:**
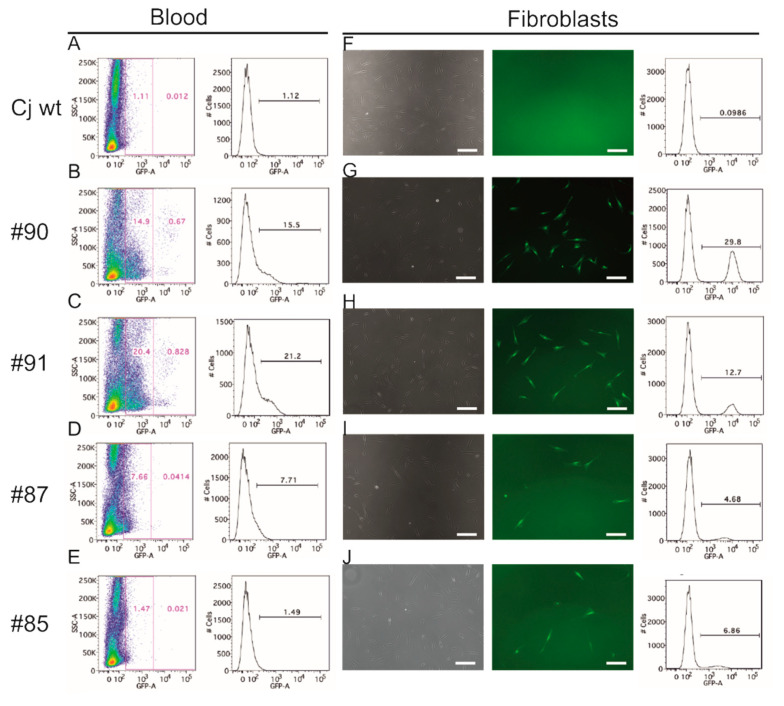
Quantification of EGFP-positive cells by flow cytometry. (**A**–**E**) Peripheral blood cells. (**A**) Wt control. (**B**–**E**) EGFP-transgenic animals #90, #91, #87, and #85, respectively. The transgenic animals show between 1.5% and 21.2% EGFP-positive cells. (**F**–**J**) Cultured skin fibroblasts. Left column: bright-field images; middle column: fluorescence images; right column: flow-cytometric quantification of EGFP-positive fibroblasts. Scale bars: 100 µm. (**F**) Wt negative control. (**G**–**J**) EGFP-transgenic animals #90, #91, #87, and #85. The fibroblast cultures from the transgenic animals show between 4.7% and 29.8% EGFP-positive cells.

Three of the resulting animals died postnatally due to an *E. coli* infection (animal cj#82), an atresia of the esophagus (cj#83), and acute circulatory failure under anesthetics after a shoulder dislocation (cj#81), respectively. The deceased animal cj#83 was transgenic, while cj#81 and #82 were not transgenic. The remaining eight animals were considered healthy without pathological findings during clinical inspection. *EGFP*-specific PCR on genomic DNA from blood cells of these eight animals showed that four of them were transgenic (cj#85, #87, #90, #91 with the two latter ones being siblings; all others were singletons; Figure 1F–I) resulting in a transgenic animal production rate (living transgenic animals/injected embryos) of 3.5% (Table 1). The founders were also genotyped by PCR on skin DNA (not shown), and results from both analyses were in accordance with each other. The rate of transgenic animals/born animals (including the three that did not survive to adulthood) was 45% (5/11). Figure 1G shows a transgenic monkey (#90) at the age of 28 days and Figure 1H the same transgenic monkey as an adult. All F0 animals genotyped as transgenic also had EGFP-positive skin fibroblasts besides non-fluorescent cells (Figure 2). Those animals in which the transgene was neither detected in blood cells nor in skin fibroblasts were classified as non-transgenic. All transgenic monkeys were macroscopically indistinguishable from the non-transgenic ones and reached ages of >6 years without any complications and signs of ill health. As control, 23 natural uninjected embryos were retransferred during 14 ET to surrogate mothers (Table 1). These 14 ET resulted in five pregnancies and birth of five animals (three singletons and one pair of twins) corresponding to a birth rate/ET of 35.7%. Four control animals grew up to adulthood without complications while one control animal died at the age of 6 months due to circulatory failure. The difference in the birth rates between both groups (injected vs. non-injected embryos) is not significantly different (*p* = 0.53).

### 3.2. Sex Ratio and Normal Postnatal Development of the Transgenic Monkeys

Male as well as female monkeys resulted from the transferred embryos (Table 2). In the transgenic group, we obtained 3 males and 1 female. The non-transgenic group, consisting of four non-transgenic animals from the injected embryos and of four control animals from the uninjected group, also showed a male:female ratio of 3:1 (six males, two females). Normal birth weight is an important indicator of normal intrauterine growth and development as well as the health of the offspring [53]. To test whether the *EGFP* transgene has an impact on the postnatal gain of body weight, we determined the postnatal body weights of the four transgenic founders with the weights of the eight non-transgenic animals that were obtained after ET. Both groups comprised one pair of twins, while all other animals were singletons, which have on average a higher birth weight than twins. There was no significant difference between the body weights in both groups at either postnatal developmental time point (Table 2) suggesting that the transgene itself does not have adverse effects reflected by an altered body weight.

### 3.3. EGFP Is Functionally Expressed in Founder Animals

After detection of the *EGFP* transgene in blood cell gDNA (Figure 1F), we were interested in whether the transgene was also expressed as a functional fluorescent protein in the founder animals. Therefore, we analyzed nucleated blood cells of the founders and a wildtype control animal by flow cytometry. In the non-transgenic control animal, the background signals comprised ~1.1% of all events (Figure 2A). In the founders, we detected between 1.5% and 21.2% EGFP-positive nucleated blood cells (Figure 2B–E). Interestingly, particularly in #90 and #91 there was a small, yet distinct population of blood cells with high fluorescence intensity. To confirm EGFP expression in a second cell type, we analyzed skin fibroblasts. The fraction of EGFP-positive fibroblasts from the transgenic animals ranged from 4.7% to 29.8% (Figure 2G–J), while the background value of wildtype fibroblasts was 0.1% (Figure 2F). These data show that functional EGFP protein is present in a subset of cells derived from two different cell lineages, i.e., skin fibroblasts and nucleated blood cells.

### 3.4. Robust EGFP Transgene Expression in Organs of a Founder Animal

**Figure 3:**
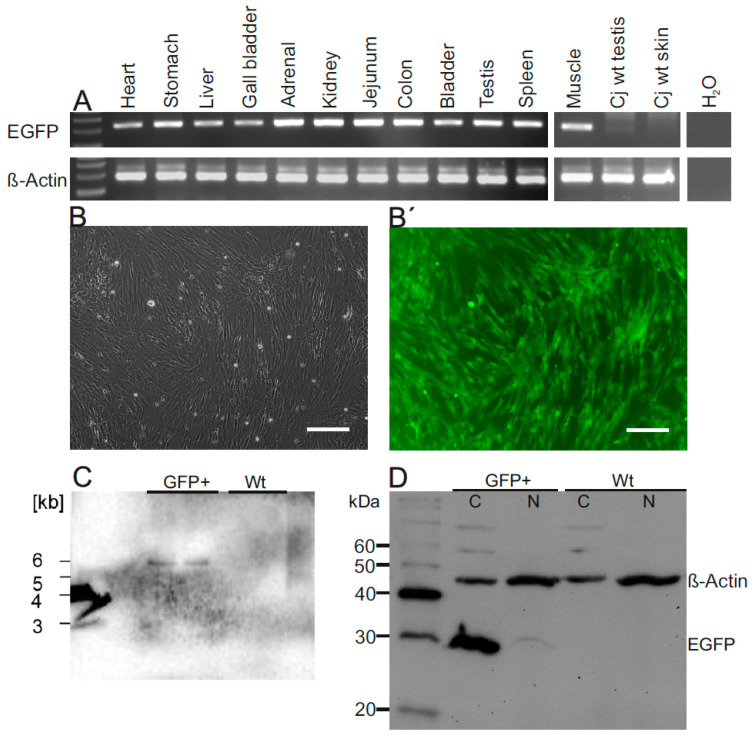
EGFP expression in the deceased neonate #83. (**A**) The transgene was detected by PCR on genomic DNA in all organs. (**B**) Bright-field image and (**B**′) fluorescence image of cultured skin fibroblasts from animal #83. Scale bars: 100 µm. (**C**) Genomic Southern blot of fibroblast DNA from #83 (GFP+) and from wt fibroblasts using an *EGFP*-specific probe. Only one band of around 6 kb is visible in the transgenic animal. Samples were loaded in duplicate. (**D**) EGFP western blot analysis of fibroblast protein from #83 (GFP+) and from wt control. From both samples cytoplasmic (C) and nuclear (N) protein fractions were analyzed. Only the cytoplasmic fraction of the cells of animal #83 showed an EGFP band.

The possibilities to analyze transgene expression in living founder animals are limited, and skin fibroblasts and blood cells are both of mesodermal origin. We took advantage of the unintended death of the neonatal animal #83 obtained from the injected embryo group and detected the transgene in gDNA of all organs tested (Figure 3A).

**Figure 4:**
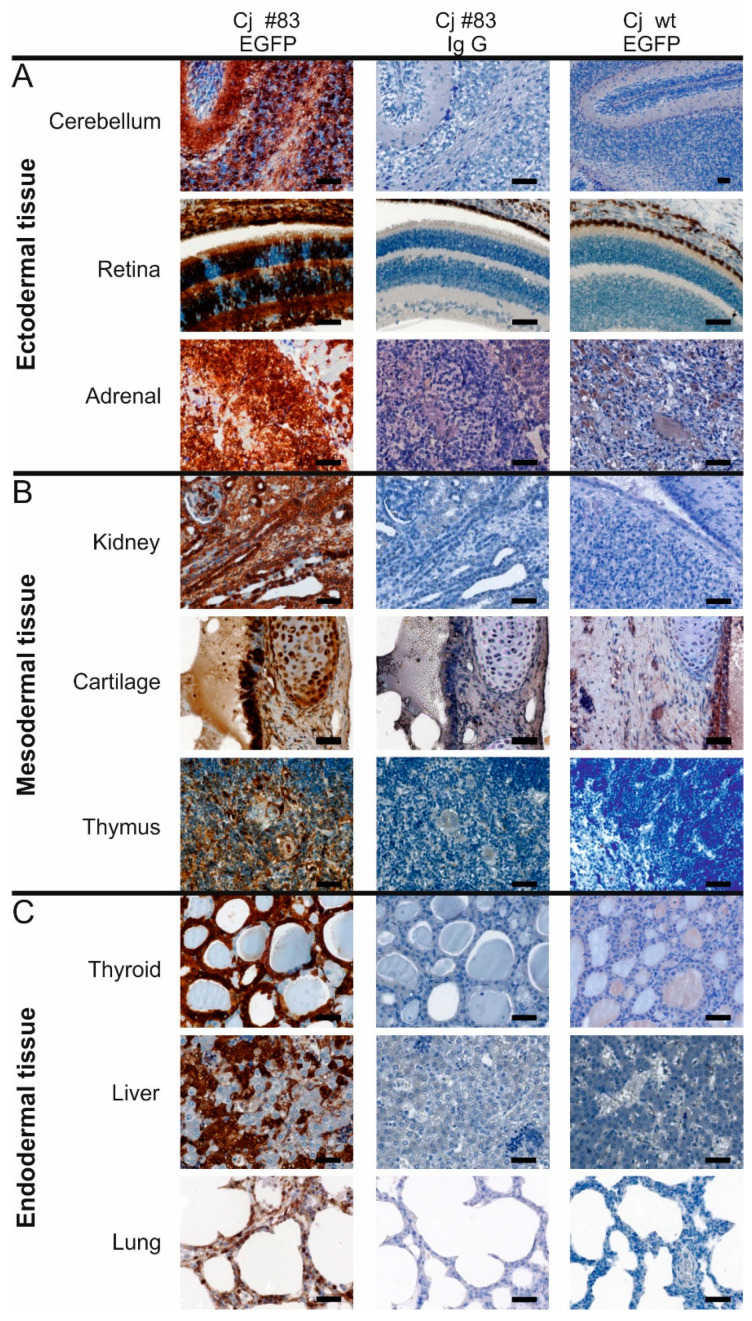
EGFP expression analysis in tissues of #83. Left column: images of tissues from the EGFP-transgenic animal #83 incubated with the EGFP antibody. Middle column: consecutive sections of the same samples incubated with the IgG control serum. Right column: corresponding wt samples incubated with the EGFP antibody. (**A**) shows tissue samples of ectodermal, (**B**) of mesodermal and (**C**) of endodermal origin. Scale bars: 50 µm.

We then performed a thorough analysis of the tissue samples from #83 (Figure 3 and Figure 4). Fluorescence microscopy of fibroblasts of cj#83 showed that almost all cells were EGFP-positive (Figure 3B,B′). Southern blot analysis of the fibroblasts indicated the presence of a single copy of the transgene (Figure 3C). Using an EGFP-specific antibody (Figure 3D), we detected robust EGFP expression in all tissues analyzed including ectodermal (Figure 4A), mesodermal (Figure 4B), and endodermal derivatives (Figure 4C). EGFP staining was also seen in all other tissues tested including heart, skeletal muscle, esophagus, and epidermis (data not shown). However, most tissues were mosaic for EGFP protein. This was particularly evident in the retina, the adrenal gland, the thyroid, and the liver (Figure 4).

### 3.5. Germline Transmission of the Transgene by Natural Mating

**Figure 5:**
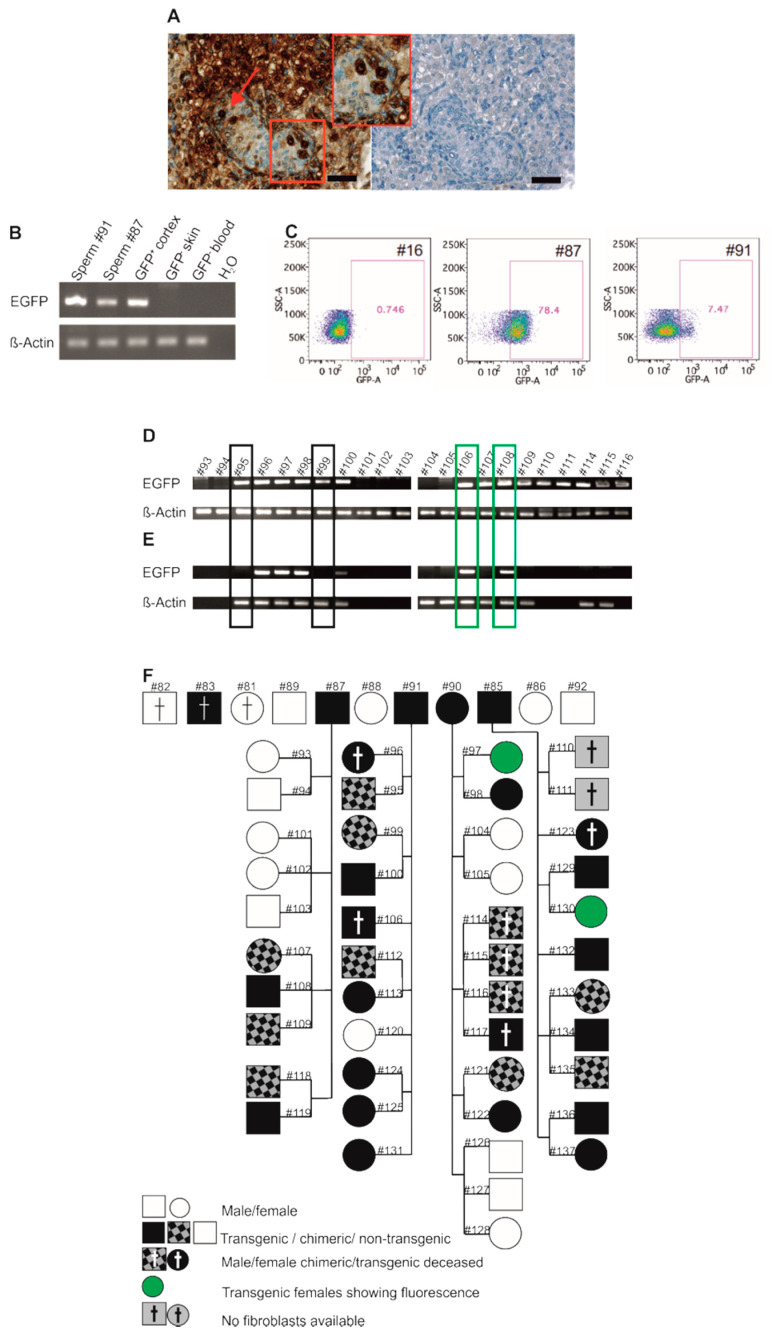
Germline transmission of the transgene. (**A**) Immunohistochemical detection of EGFP in the neonatal testis of #83. All germ cells show an intense EGFP signal, while intra-tubular somatic cells show only little EGFP signals. Interstitial somatic cells are strongly stained. The boxed area is shown at higher magnification (middle image in (**A**)). Right image: corresponding negative (wt) control. Scale bars: 50 µm. (**B**) *EGFP* PCR on genomic DNA isolated from ejaculated sperm from transgenic founders #87 and #91. Both samples show *EGFP* amplicons. (**C**) Flow-cytometric analysis of semen samples from transgenic founders #87 and #91 and a wt control (#16). The transgenic samples show fluorescence above background levels. (**D**) Genotyping of an exemplary subset of F1 progeny using DNA directly isolated from skin. β-Actin was used as positive control. The genotyping results for all F1 animals are shown in Figure S1. (**E**) Genotyping of F1 progeny using DNA isolated from frozen-thawed and re-cultured skin fibroblasts. Only a subpopulation of the animals being positive in (**D**) remained *EGFP*-positive using selected fibroblasts (two animals exemplarily highlighted by green boxes), while chimeric littermates of transgenic animals switched from “positive” to “negative” (two animals exemplarily highlighted by black boxes). β-Actin was used as a positive control. Those animals that show no β-Actin band in (**E**) (#110 and #111) were not tested using hematopoietic cell-depleted cell cultures. (**F**) Pedigree showing the founders and 45 F1 animals obtained from natural mating of animals #87, #91, #90, and #85 with wt partners (latter not depicted). All founders were fertile and transmitted the transgene to progeny.

Genetic mosaicism occurs when an organism, which developed from a single zygote, consists of two or more populations of cells with different genotypes. To obtain non-mosaic, ubiquitously transgene expressing monkeys, transduction of germline cells and germline transmission of the transgene to the offspring are required, if cloning should be avoided. Besides in gonadal somatic cells, we also detected strong EGFP protein expression in the neonatal testicular germ cells (gonocytes) of #83 by immunohistochemistry (Figure 5A).

Three founders (#85, #87, #91) in our study were males (Figure 1I). Two of them (#87, #91) donated ejaculates, and the respective DNA samples were positive for *EGFP* (Figure 5B). We also tested spermatozoa from these founders for EGFP by flow cytometry and detected 78.4% (#87) and 7.5% (#91) of the cells, respectively, in the gate representing EGFP-positive cells, while the wildtype control (#16) showed background events in 0.7% of all spermatozoa (Figure 5C). This suggests that functional EGFP is present in at least a subset of ejaculated spermatozoa. However, particularly in animal #87, the whole sperm population was shifted to increased signal intensity, which contrasts with the signal distribution pattern seen with other cell types such as fibroblasts, which show two clearly separated cell populations (see Figure 2). The observed shift of the whole spermatozoa population as seen in animal #87 is most likely due to the fact that the genetically distinct postmeiotic haploid spermatozoa progenitors are functionally diploid [54], i.e., they share the gene products of their haploid genomes via intercellular cytoplasmic bridges connecting a large number of the spermatids. Hence, the spermatozoa can be phenotypically similar despite their genetic heterogeneity. Nevertheless, this does not exclude that different levels of EGFP are present in individual spermatozoa and may explain the lack of two clearly separated cell populations.

When we mated the four founders to wildtype marmosets, all produced offspring. Altogether 5 singletons, 12 twin pairs, 4 triplets, 1 quadruplet were obtained. However, one twin pair [#110 and #111] could not be correctly genotyped and was therefore excluded from subsequent calculations. The transgene was detected by PCR in primary cell cultures derived from skin samples and in blood cell samples in 32 out of 43 F1 animals (74.4%) suggesting highly efficient germ line transmission of the transgene by natural mating (Figure 5D for exemplary samples and Figures S1A and S2 for the whole set of animals). However, litter-wise analysis of the inheritance of the transgene revealed that it was detectable either in all offspring or in none (Figure 5D, Figures S1A and S2).

### 3.6. Genotyping of Transgenic Offspring Reveals Consistent Cell Chimerism in Siblings

We wondered whether cell chimerism could account for this transgene detection pattern in the F1 litters. A chimeric organism consists of cells from different zygotes. In marmosets, blood cell chimerism occurs due to the exchange of hematopoietic stem cells through placental blood vessel anastomoses between siblings [55,56]. Moreover, it is still disputed whether other organs than the hematopoietic system are also chimeric in marmosets [57,58,59]. Therefore, we cultured skin fibroblasts of the F1 animals (except #110 and #111, which were not available for fibroblast analysis) for at least 5 days after a freezing/thawing cycle to deplete the fibroblast cultures of potentially contaminating blood-derived (including chimeric) immune cells. When we genotyped these immune cell-depleted fibroblasts we obtained significantly different results compared to the data obtained from blood and native skin samples: 20 out of 43 tested F1 animals (46.5%; instead of 32 out of 43 corresponding to 74.4%) were transgenic (Figure 5E,F, Figure S1B; for a full list of F1 animals see also Supp. Table S1). Twelve F1 animals that initially tested transgenic showed no *EGFP* signal in post-freezing fibroblast cultures (Figure 5E,F and Figure S1) showing that the fibroblast freezing and re-culturing successfully removed contaminating chimeric cells. The validity of the removal procedure was confirmed by a PCR for *SRY*, which is a male-specific Y-chromosomal gene, on selected samples from mixed-sex siblings. All treated fibroblast cultures from phenotypically male animals exhibited *SRY*, while fibroblasts from phenotypically female animals did not show *SRY*, even if the female had a male co-twin (Figure S3; #99 and #100, #107–#109, #112 and #113). In conclusion, these data show that the transgene was transmitted to offspring by all founders by natural mating. This demonstrates that transgenic gametes are competitive in the complex process of in vivo sperm selection and natural fertilization. Our data also indicate that cell chimerism between siblings occurs regularly and not only occasionally. Furthermore, our data confirm that the material used for genotyping of (GM) marmosets must be carefully selected to prevent misleading results, e.g., tips of fingernails [33,59] or carefully prepared skin samples.

### 3.7. Phenotyping of Progeny

**Figure 6:**
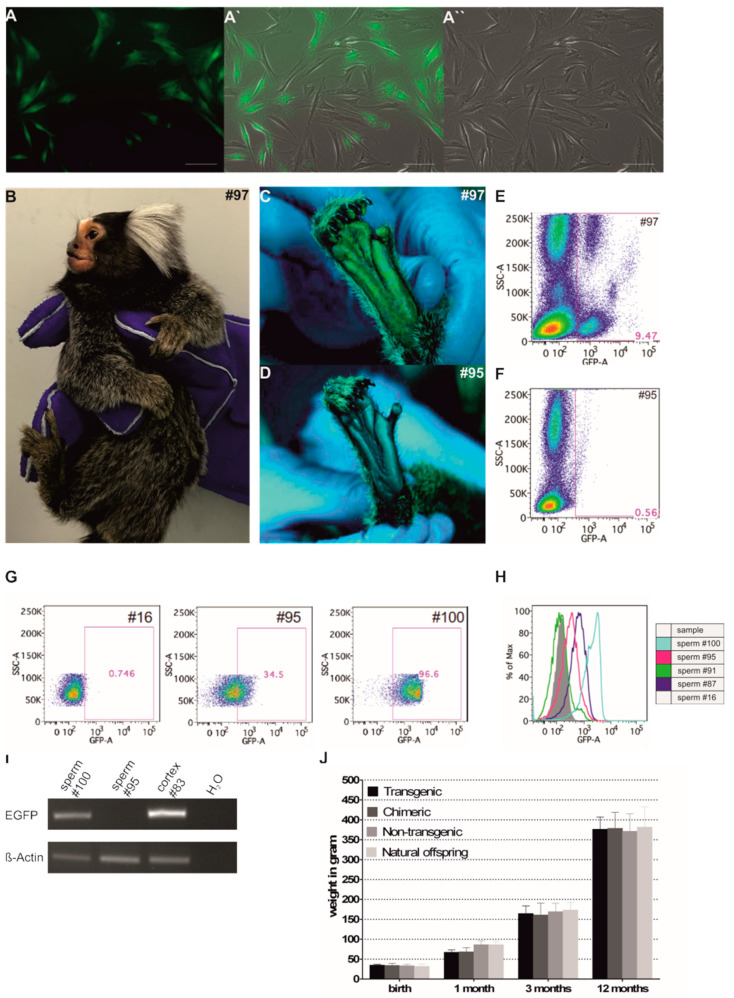
Phenotyping of the F1 animals. (**A**) Cultured fibroblasts of animal #97 showing EGFP fluorescence. A: Fluorescence image, (**A′**): Merge with (**A″**) (right panel, bright-field image). Scale bars: 50 µm. (**B**) Whole body view of transgenic F1 animal #97. (**C**) Epifluorescent image of the foot pad of transgenic #97. (**D**) Epifluorescent image of the foot pad of chimeric #95, which showed no EGFP fluorescence. (**E**) Flow cytometry of nucleated peripheral blood cells of #97 and (**F**) #95. (**G**) Flow-cytometric analyses of sperm cells of #16 (wt control), #95 (F1 chimeric), and #100 (F1 transgenic). In animal #100 the whole cell population is homogenously shifted to higher fluorescence signals with 96.6% in the gate representing EGFP-positive cells. The analyses of spermatozoa shown in Figure 5C and Figure 6G were performed in one run and the analysis of wildtype animal #16 is shown as a control in both figures for the purpose of clarity. (**H**) Histogram overlay from flow-cytometric sperm cell analyses of transgenic founders #87 and #91 and F1 animals #95 and #100. The grey filled histogram represents wt sperm (#16). (**I**) PCR genotyping of ejaculated sperm from #95 and #100. (**J**) Postnatal gain of body weight in the different study groups.

To test whether the transgenic F1 animals also express functional EGFP protein, we isolated skin fibroblasts. Fluorescence microscopy revealed only in two fibroblast samples EGFP signals (#97 and #130, indicated by a filled green circle in Figure 5F; Figure 6A,A′,A″). These animals showed normal morphology (e.g., #97; Figure 6B) and apparently normal behavior and locomotion in their experimental environment (video recordings; data not shown).

Fibroblasts of all other transgenic animals did not show fluorescence (data not shown). To corroborate EGFP expression in the animal, we performed vital fluorescence imaging of hairless skin for EGFP. Exemplarily, animals #97 (transgenic) and animal #95 (chimeric) are shown in Figure 6C,D, respectively. Although animal #97 clearly showed EGFP fluorescence, e.g., in the sole of the foot, no fluorescence was detectable in the sole of the foot of animal #95. Flow cytometry of blood cells of animals #97 and #95 further confirmed a clear difference in the EGFP signals between these F1 animals (Figure 6E,F). Semen samples of two males of the progeny (#95 and #100) were obtained and analyzed by flow cytometry in the same run in parallel with the control animal sample (#16) and the samples of the founders as shown in Figure 5C. Animal #100 showed 96.6% and animal #95 34.5% of the spermatozoa in the gate representing EGFP-positive cells (Figure 6G). As already seen for #87 (Figure 5C), the whole population of spermatozoa was shifted towards increased fluorescence with clear differences between the average fluorescence intensities of the spermatozoa from animals #87 (founder), #91 (founder), #95 (chimeric F1), and #100 (transgenic F1) (Figure 6H). Importantly, however, when we analyzed spermatozoa DNA from animals #95 and #100 by PCR, only #100 showed an *EGFP* signal, while #95 was negative (Figure 6I). We conclude that the EGFP transgene is inactive in fibroblasts of all F1 progeny except animals #97 and #130. However, F1 animal #100 clearly showed *EGFP* signals by PCR and by flow cytometry in spermatozoa. The reasons for increased fluorescence of spermatozoa from #95 currently remain speculative, but may involve EGFP deposition on stored spermatozoa by chimeric epididymal immune cells (the sibling of #95, #96, was transgenic) or by the seminal fluid, both of which contain mast cells [60].

As for the founder generation, we also monitored the birth weights and the postnatal gain of body weight in the F1 generation. To avoid a misleading influence of the weight of singletons, they were excluded from the analysis. The average birth weight of the transgenic offspring was neither significantly different from the birth weights of the chimeric animals nor from the non-transgenic F1 animals from this study, nor from an external reference group from the general DPZ breeding colony (Figure 6J). Although there were slight differences in the average body weights at the subsequent time points, there was also no significant difference between the groups. We conclude that (1) the transgenic F1 animals generally develop normally, but also that (2) the expression of the transgene varies in the F1 progeny with functional inactivation of the transgene in the fibroblasts of most animals.

## 4. Discussion

Due to their close phylogenetic relationship to humans, NHP are advantageous species to model aspects of human physiology and pathology [1], and there is an increasing demand to develop NHP as meaningful in vivo models of human diseases [2,15,16,17,18,19,20,21,33,34,35,36,37,61]. A promising approach to achieve modeling of many human diseases is genetic modification of NHP [62]. Nuclease-mediated inactivation and modification of genes has been successfully used in NHP [15,16,61,63,64,65,66,67]. Yet, for the overexpression of pathogenic proteins [33,34,36,37] or of in vivo reporter genes [40] lentiviral expression systems are still attractive alternatives, which have also been established in rodents and other mammals [28,68]. However, it is necessary to gain more species-specific knowledge about transgenesis in NHP to efficiently generate and appropriately assess NHP disease models.

In the present study, we used naturally conceived multicellular embryos for the introduction of the transgene, since natural embryos have a better developmental potential compared to in vitro generated marmoset embryos [29]. The disadvantage of multicellular embryos compared to one cell-stage embryos, i.e., the in vitro fertilized oocyte, during genetic modification is that this approach leads to genetic mosaicism in the modified embryos. Although modifications at the one cell stage can result in a homogenous modification of all cells of the resulting animal [63], it is still necessary for most purposes to breed or, alternatively, clone [69] the GM founders to generate a homogenous population of GM NHP. Remarkably, all four EGFP-transgenic founder animals in our study produced transgenic offspring when naturally mated to wildtype marmosets. So far, there are relatively few reports on progeny from GM NHP, e.g., [29,33,34,36,39], and the number of F1 animals was rather limited in these studies. In the present study we provide an analysis of 22 F1 litters including 20 transgenic, 12 chimeric, and 11 wildtype offspring plus two pups of unclear genotype. However, at least one of the latter was also transgenic. Transgene transmission through the female germline by natural mating has been reported [33]. However, to the best of our knowledge, we report here for the first time germline transmission through the male NHP germline by natural mating. Oogenesis and spermatogenesis, the processes resulting in the production of mature eggs and spermatozoa, respectively, are strictly quality controlled, resulting in the release of highly selected mature gametes [70,71]. Furthermore, after natural mating only a very small, yet again highly selected population of the millions of ejaculated sperm gain access to the site of fertilization, i.e., the ampulla of the oviduct [72]. Using ART and in particular ICSI, the selection of the potentially fittest and “healthiest” sperm is overridden. Considering further that natural spermatozoa are heterogenic [73,74], the use of ICSI may result in fertilization of the oocyte by spermatozoa that would not succeed in natural fertilization [72]. In fact, there is evidence that ART-derived offspring differs from natural offspring [75,76,77,78,79]. Therefore, we consider it important that Tomioka and colleagues [33] and our present study demonstrate successful natural breeding of transgenic marmosets as an alternative to ART. On the other hand, it is undoubted that well-established ART procedures [80] are indispensable for specific purposes of marmoset biotechnology and disease model generation.

We used the FUGW lentiviral vector, in which the ubiquitous human *UBC* promoter drives *EGFP* expression. This vector/promoter combination was previously shown to be reliable and robust in vivo in mice [28,48], rats [28], and macaques [37]. In the present study, all four F0 founders showed fluorescence in diverse cell types, and the deceased neonate (#83) expressed high levels of EGFP in all analyzed tissues, indicating that the *UBC* promoter generally also works robustly in F0 marmoset monkeys. Remarkably, the fibroblasts from #83 showed very strong fluorescence although the cells carried only one copy of the transgene. In a previous study, EGFP fluorescence was visible only in mice with at least two copies of the transgene [28] while in macaques [37] EGFP fluorescence was also detected in single copy transgenic F0 animals suggesting that the human *UBC* promoter may work more efficiently in F0 NHP than in F0 mice. In sharp contrast to the F0 marmosets in our study, where five out of five transgenic animals expressed EGFP, only two out of 20 transgenic F1 progeny showed fluorescence in cultured fibroblasts. This suggests that the human *UBC* promoter-driven vector is frequently silenced during progression through the NHP germline, which involves extensive epigenetic reprogramming [81,82]. It is striking that in contrast to the marmoset, the FUGW vector was generally not silenced during germline transmission in rodents [28]. We speculate that this may reflect different defense mechanisms against foreign DNA in the germline of short-living rodents and long-living primates [83]. However, the actual reasons for the lack of detectable EGFP in most F1 marmosets and whether there are indeed primate-specific mechanisms of lentiviral transgene silencing remain to be analyzed. We conclude that specific transgenic marmoset monkey lines need to be carefully analyzed and selected before being used in further applications. In general, transgenesis is, due to the high germline transmission rates, a straightforward approach for the generation of NHP disease lines. An attractive alternative to lentiviral transgenesis, however, could be transposon-based transgenesis, which was recently used by us to generate GM rodents and ungulates and proved to be very robust [84,85].

Initially, when we genotyped the F1 monkeys on blood or native skin DNA, we observed an unexpected transgene inheritance pattern with the transgene either being present in all pups of a litter or in none. Based on our analyses, we conclude that the native samples were chimeric. In fact, all F1 monkeys that had a transgenic sibling were initially also genotyped as transgenic, and only after cell selection they turned out to be actually wildtype thereby also revealing the presence of genotypically mixed litters. Marmosets typically produce dizygotic twins (or trizygotic triplets), i.e., the embryos are genetically non-identical in contrast to mono-zygotic twins. Although developing from different embryos, the placentas of the marmoset embryos usually fuse transiently and form anastomoses resulting in a shared blood circulation (monochorionic twinning). It is known that this eventually results in a significant blood stem cell chimerism in *Callitrichidae* [55,86,87] without the manifestation of the free-martin syndrome found in male-female twins of cattle [55]. Our data suggest that blood cell chimerism in marmosets occurs invariably and not only occasionally. However, it is still a matter of debate if other organs also exhibit cell chimerism in marmosets. Although one study suggested this [57], others suggested that the findings of chimerism in solid organs are rather the result of blood or lymphocytic infiltration of these organs instead of a primary chimerism [87]. Our findings using the selected fibroblasts indicate that they are not chimeric. This is supported by the fact that the male-specific *SRY* gene was not detectable in selected fibroblasts of females being the fraternal twin of a male. We also tested the ejaculates of two F1 males and detected *EGFP* only in one truly transgenic animal but not in a chimeric animal. Hence, in two cell populations, i.e., fibroblasts and spermatozoa, derived from chimeric animals, we were not able to detect *EGFP* by PCR undermining the view that chimerism is present also in non-bone-marrow-derived cells. Our study rather supports the view that the detected chimerism in marmosets is based on lymphocytic infiltration and not on tissue-specific cell chimerism. This view is also strongly supported by studies using fingernail DNA for genotyping [33,59]. The transgene-chimeric littermates obtained in our study may represent a valuable resource for chimerism studies in marmosets.

Macaques usually deliver singletons, and natural twins are rare. Only 4 pairs of twins were found after natural conception among 1748 rhesus macaque births [88]. This incidence is much lower than in humans and in sharp contrast to marmosets. Importantly, however, in almost all studies on GM macaques, multiple embryos were transferred to surrogate mothers [32,37,61,64,65,89], and in almost all studies multiple pregnancies, often associated with severe problems including abortions and stillbirths, have been observed. Nevertheless, several studies also reported the birth of live twins [37,64,65,89] and even triplets [65]. Therefore, it would be highly expedient to carefully test whether (blood) cell chimerism can also occur in macaques. This is important considering that the limited information on the placental development in macaque twin pregnancies does not exclude that placental fusion, with the possibility of an exchange of hematopoietic stem cells, may also occur in macaques [90,91].

## 5. Conclusions

In conclusion, we have shown that the lentiviral transgene is efficiently transmitted to offspring by natural mating. However, in contrast to rodents, the *UBC* promoter-driven transgene is frequently silenced in F1 monkeys. Therefore, alternative promoters or expression systems should be considered in future studies. We consistently observed (blood) cell chimerism in F1 animals from multiple pregnancies. Therefore, for the practical work with GM marmosets, particularly in the field hematopoiesis and immunology, it is important to take this specific biological characteristic of marmosets into consideration. However, carefully selected transgenic NHP lines will be useful to investigate primate physiology and to model aspects of human pathology.

## Figures and Tables

**Table 1:** Production rate of *EGFP*-transgenic marmosets.

KERRYPNX	Virus Injection	Controls (Not Injected)
Embryos used	113	23
Embryos transferred	110	23
Number of embryo transfers (ET)	46	14
Embryos transferred per ET	1–4	1–3
Average number of embryos per ET	2.4	1.6
Pregnancies	14	5
Births	11	5
Birth rate (Birth per ET)	23.9%	35.7%
Animals alive	8	4
Number of vital transgenic animals (tg)	4	n.a.
Number of deceased transgenic animal	1	n.a.
Production rate of vital animals (tg per injected embryos)	3.5%	n.a.

**Table 2:** Postnatal gain of weight of marmosets after embryo transfer.

	Transgenic		Non-Transgenic	
Number of animals	4		8	
Ratio ♂:♀	3:1		3:1 (6/2)	
	mean	range	mean	range
weight at birth (g)	39.2	36.6–44.7	35.7	33.0–39.0
weight at 1 month	66.2	60.4–76.2	65.3	44.6–80.0
weight at 3 months	171	149–180	169.4	145–200
weight at 12 months	359	312–433	378.5	288–450

## Data Availability

Data is contained within the article or supplementary material.

## Supplementary Materials

The following are available online at https://www.mdpi.com/2073-4409/10/3/505/s1, Figure S1: Genotyping of F1 progeny using DNA directly isolated from skin, Figure S2: Pedigree showing the founders and 45 F1 animals according to genotyping of F1 progeny using DNA directly isolated from skin, Figure S3: *SRY* genotyping of DNA from freezing-thawing-selected fibroblasts, Table S1: Overview and dates of births of the F1 generation.
