# Supplementary material for: Generation and Breeding of EGFP-Transgenic Marmoset Monkeys: Cell Chimerism and Implications for Disease Modeling
Source: Cells. 2021 Feb 27;10(3):505. doi: 10.3390/cells10030505 (PMC7996964; doi:10.3390/cells10030505)
Supplement: Supplementary file 1 [file cells-10-00505-s001.zip › Supplementary/Suppl. Fig.1_final_Drummer et al..pdf]

Drummer et al., Suppl. Figure 1

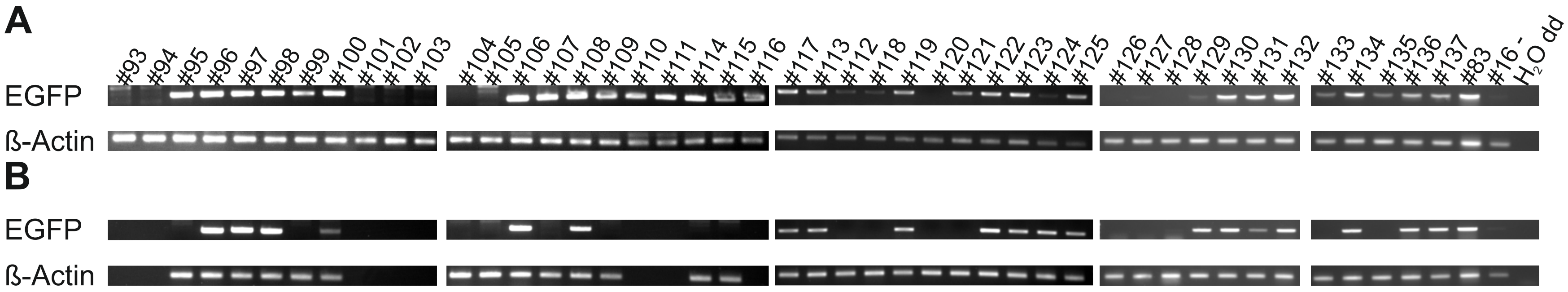

A) *Genotyping of F1 progeny using DNA directly isolated from skin. β-Actin was used as positive control.* ***B)*** *Genotyping of F1 progeny using DNA isolated from frozen-thawed and re-cultured skin fibroblasts. Only a subpopulation of the animals being positive in A) remained EGFP-positive using selected fibroblasts, while chimeric littermates of transgenic animals switched from "positive" to "negative". β-Actin was used as positive control. Those animals that show no β-Actin band in B) (#110 and #111) were not tested using hematopoietic cell-depleted cell cultures.*
