# Supplementary material for: Generation and Breeding of EGFP-Transgenic Marmoset Monkeys: Cell Chimerism and Implications for Disease Modeling
Source: Cells. 2021 Feb 27;10(3):505. doi: 10.3390/cells10030505 (PMC7996964; doi:10.3390/cells10030505)
Supplement: Supplementary file 1 [file cells-10-00505-s001.zip › Supplementary/Suppl. Fig.2_final_Drummer et al..pdf]

Drummer et al., Suppl. Figure 2

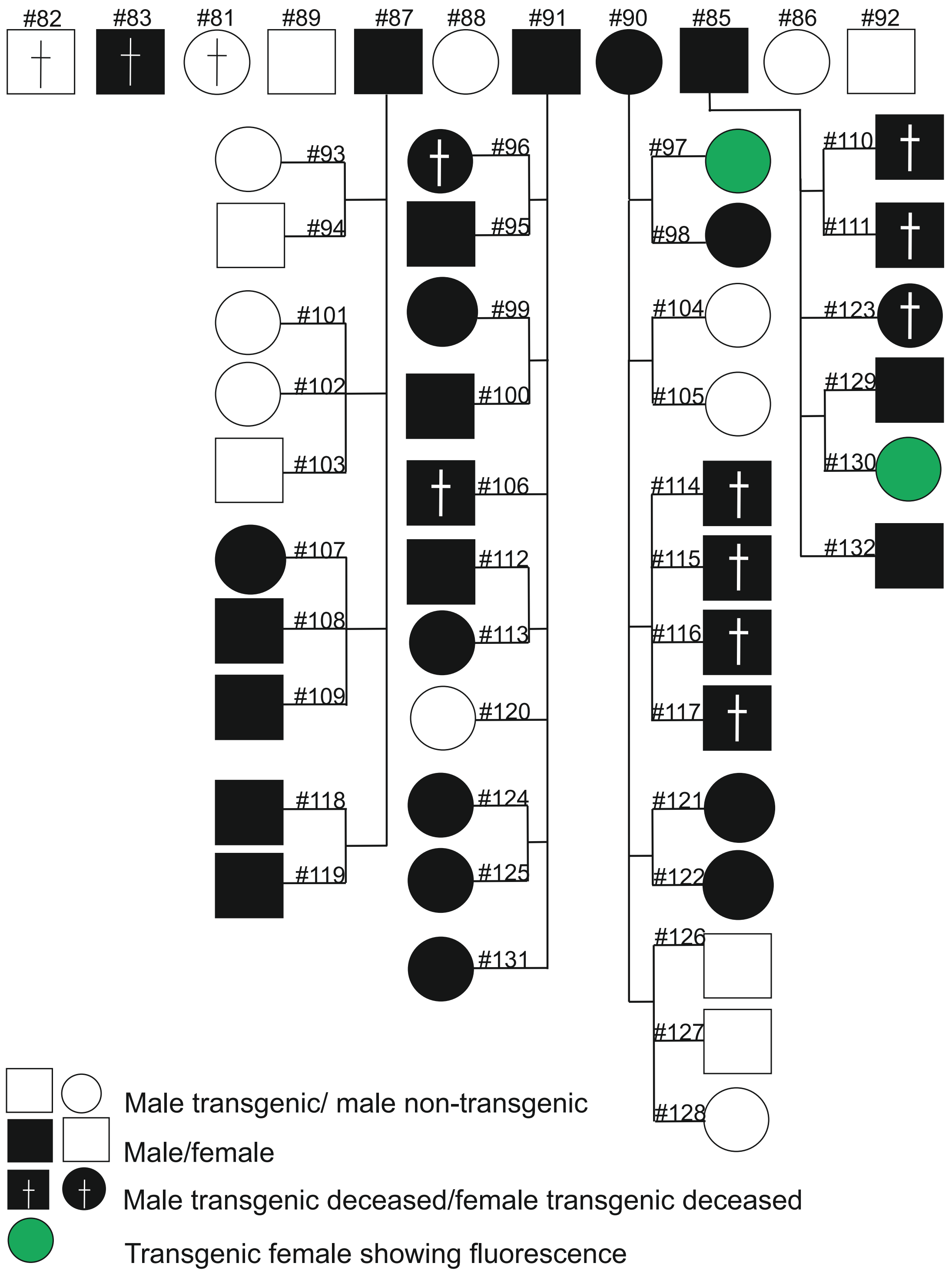

Pedigree showing the founders and 45 F1 animals obtained from natural matings of animals #87, #91, #90, and #85 with wt partners (latter not depicted). Genotyping was performed before chimeric cell depeletion on native cultured skin cell samples.
