# Supplementary material for: Generation and Breeding of EGFP-Transgenic Marmoset Monkeys: Cell Chimerism and Implications for Disease Modeling
Source: Cells. 2021 Feb 27;10(3):505. doi: 10.3390/cells10030505 (PMC7996964; doi:10.3390/cells10030505)
Supplement: Supplementary file 1 [file cells-10-00505-s001.zip › Supplementary/Suppl. Fig.3_final_Drummer et al_corrected.pdf]

Drummer et al., Suppl. Fig. 3

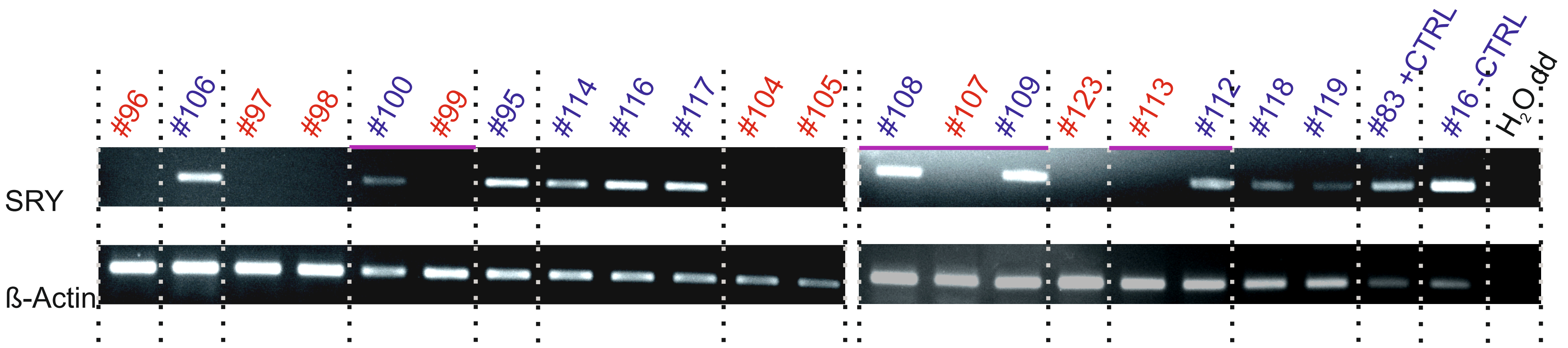

Blue number: ♂
Red number: ♀
Vertical dotted lines separate litters
Purple horizontal lines highlight mixed litters

Legend to Supp. Fig. 3: SRY genotyping indicates the lack of contaminating chimeric DNA in DNA from freezing-thawing-selected fibroblasts. SRY is a male-specific gene on the Y chromosome. Only fibroblast DNA samples from phenotypically male monkeys showed SRY signals using selected fibroblast DNA, while the corresponding samples from female littermates of male monkeys (e.g. #99 and #107) lacked SRY indicating the complete depletion of chimeric DNA during fibroblast selection. These data validate the *EGFP* genotyping data shown in Fig. 5E.
