# Supplementary material for: Generation and Breeding of EGFP-Transgenic Marmoset Monkeys: Cell Chimerism and Implications for Disease Modeling
Source: Cells. 2021 Feb 27;10(3):505. doi: 10.3390/cells10030505 (PMC7996964; doi:10.3390/cells10030505)
Supplement: Supplementary file 1 [file cells-10-00505-s001.zip › Supplementary/Suppl. Table 1_final_Drummer et al.pdf]

Suppl. Table 1: Overview and dates of births of the F1 generation

Number #	*EGFP*	Sex	Birth date (yyyy-mm-dd)	Comment
**F1 of Founder #87**				
93	negative	female	2014-08-21	
94	negative	male	2014-08-21	
101	negative	female	2015-04-30	
102	negative	female	2015-04-30	
103	negative	male	2015-04-30	
107	chimeric	female	2015-09-30	
108	positive	male	2015-09-30	
109	chimeric	male	2015-09-30	
118	chimeric	male	2016-03-27	
119	positive	male	2016-03-27	
**F1 of Founder #91**				
95	chimeric	male	2014-11-20	
96	positive	female	2014-11-20	stillbirth
99	chimeric	female	2015-04-25	
100	positive	male	2015-04-25	
106	positive	male	2015-09-28	strangulation of leg during birth
112	chimeric	male	2016-03-01	
113	positive	female	2016-03-01	
120	negative	female	2016-08-02	
124	positive	female	2017-01-04	
125	positive	female	2017-01-04	
131	positive	female	2017-06-06	
**F1 of Founder #90**				
97	positive	female	2015-04-03	
98	positive	female	2015-04-03	
104	negative	female	2015-09-05	
105	negative	female	2015-09-05	
114	chimeric	male	2016-03-19	stillbirth
115	chimeric	male	2016-03-19	stillbirth
116	chimeric	male	2016-03-19	stillbirth
117	positive	male	2016-03-19	stillbirth
121	chimeric	female	2016-08-20	
122	positive	female	2016-08-20	
126	negative	male	2017-02-04	
127	negative	male	2017-02-04	
128	negative	female	2017-02-04	
**F1 of Founder #85**				
110	positive	male	2016-01-24	stillbirth
111	positive	male	2016-01-24	stillbirth
123	positive	female	2016-09-20	dead after c-section
129	positive	male	2017-06-29	
130	positive	female	2017-06-29	
132	positive	male	2018-02-28	
133	chimeric	female	2018-08-01	
134	positive	male	2018-08-01	
135	chimeric	male	2018-08-01	
136	positive	male	2019-01-02	
137	positive	female	2019-01-02	
